# A Dual‐Channel Deep Learning Approach for Lung Cavity Estimation From Hyperpolarized Gas and Proton MRI

**DOI:** 10.1002/jmri.28519

**Published:** 2022-11-14

**Authors:** Joshua R. Astley, Alberto M. Biancardi, Helen Marshall, Paul J. C. Hughes, Guilhem J. Collier, Laurie J. Smith, James A. Eaden, Rod Hughes, Jim M. Wild, Bilal A. Tahir

**Affiliations:** ^1^ POLARIS, Department of Infection, Immunity & Cardiovascular Disease The University of Sheffield Sheffield UK; ^2^ Department of Oncology and Metabolism The University of Sheffield Sheffield UK; ^3^ Early Development Respiratory AstraZeneca Cambridge UK; ^4^ Insigneo Institute for in silico medicine, The University of Sheffield Sheffield UK

**Keywords:** deep learning, segmentation, pulmonary MRI, hyperpolarized gas MRI

## Abstract

**Background:**

Hyperpolarized gas MRI can quantify regional lung ventilation via biomarkers, including the ventilation defect percentage (VDP). VDP is computed from segmentations derived from spatially co‐registered functional hyperpolarized gas and structural proton (^1^H)‐MRI. Although acquired at similar lung inflation levels, they are frequently misaligned, requiring a lung cavity estimation (LCE). Recently, single‐channel, mono‐modal deep learning (DL)‐based methods have shown promise for pulmonary image segmentation problems. Multichannel, multimodal approaches may outperform single‐channel alternatives.

**Purpose:**

We hypothesized that a DL‐based dual‐channel approach, leveraging both ^1^H‐MRI and Xenon‐129‐MRI (^129^Xe‐MRI), can generate LCEs more accurately than single‐channel alternatives.

**Study Type:**

Retrospective.

**Population:**

A total of 480 corresponding ^1^H‐MRI and ^129^Xe‐MRI scans from 26 healthy participants (median age [range]: 11 [8–71]; 50% females) and 289 patients with pulmonary pathologies (median age [range]: 47 [6–83]; 51% females) were split into training (422 scans [88%]; 257 participants [82%]) and testing (58 scans [12%]; 58 participants [18%]) sets.

**Field Strength/Sequence:**

1.5‐T, three‐dimensional (3D) spoiled gradient‐recalled ^1^H‐MRI and 3D steady‐state free‐precession ^129^Xe‐MRI.

**Assessment:**

We developed a multimodal DL approach, integrating ^129^Xe‐MRI and ^1^H‐MRI, in a dual‐channel convolutional neural network. We compared this approach to single‐channel alternatives using manually edited LCEs as a benchmark. We further assessed a fully automatic DL‐based framework to calculate VDPs and compared it to manually generated VDPs.

**Statistical Tests:**

Friedman tests with post hoc Bonferroni correction for multiple comparisons compared single‐channel and dual‐channel DL approaches using Dice similarity coefficient (DSC), average boundary Hausdorff distance (average HD), and relative error (XOR) metrics. Bland–Altman analysis and paired *t*‐tests compared manual and DL‐generated VDPs. A *P* value < 0.05 was considered statistically significant.

**Results:**

The dual‐channel approach significantly outperformed single‐channel approaches, achieving a median (range) DSC, average HD, and XOR of 0.967 (0.867–0.978), 1.68 mm (37.0–0.778), and 0.066 (0.246–0.045), respectively. DL‐generated VDPs were statistically indistinguishable from manually generated VDPs (*P* = 0.710).

**Data Conclusion:**

Our dual‐channel approach generated LCEs, which could be integrated with ventilated lung segmentations to produce biomarkers such as the VDP without manual intervention.

**Evidence Level:**

4.

**Technical Efficacy:**

Stage 1.

Respiratory diseases are among the leading causes of mortality and disability worldwide.^1^ Imaging plays an important role in the diagnosis, treatment planning, monitoring, and treatment assessment of respiratory diseases.^2^, ^3^, ^4^ Computed tomography (CT) is the reference standard in clinical practice for most patients with respiratory diseases.^5^ Recent advances in proton MRI (^1^H‐MRI) have overcome historical challenges in using this modality for pulmonary imaging, including the low proton density and many air–tissue interfaces in the lungs.^6^ Despite the strengths of both these modalities, they only provide structural information and not information on regional lung function. Hyperpolarized gas MRI has shown applicability for functional lung imaging including lung ventilation quantification,^7^ treatment response assessment,^8^ and for functional lung avoidance radiotherapy.^9^ Hyperpolarized gas MRI enables quantification of regional lung ventilation with high spatial and temporal resolution,^10^ allowing the computation of clinical biomarkers such as the ventilation defect percentage (VDP).^7^, ^11^

The VDP is computed from segmentations derived from spatially co‐registered, hyperpolarized gas MRI and structural ^1^H‐MRI.^12^ To ensure spatial alignment, both modalities are acquired consecutively and at approximately the same lung inflation level. However, the acquired scans are frequently misaligned, given that image registration, which assumes topology preservation between fixed and moving images, consistently underperforms in cases with large discrepancies in topology between functional and structural modalities.^13^ Consequently, the misaligned structural region of interest (the lung cavity) required for the computation of VDP poses considerable segmentation challenges. To ensure the most accurate results, particularly in cases with substantial discrepancies in inflation levels during image acquisition, a lung cavity estimation (LCE) representing the thoracic cavity volume in the spatial domain of hyperpolarized gas MRI is required. To date, no algorithm exists to automatically segment this structure and manual editing is time‐consuming.

Deep learning (DL) has shown promise for numerous pulmonary image segmentation problems.^14^ A recent review of DL applications in lung image analysis showed that the vast majority of DL lung segmentation studies employed CT.^15^ The authors identified that MRI is underrepresented in DL lung segmentation applications and thus represents a gap in the literature. In the field of DL, convolutional neural networks (CNNs) have become dominant for lung image segmentation due to their ability to accurately segment various structures with computational efficiency.^15^ Several investigators have evaluated the use of CNNs for pulmonary MRI segmentations.^16^, ^17^ Tustison et al used a three‐dimensional (3D) UNet CNN to produce ^1^H‐MRI whole‐lung segmentations, achieving a mean Dice similarity coefficient (DSC) of 0.94.^16^ Zha et al used a two‐dimensional (2D) UNet to successfully segment ultra‐short echo time (UTE) ^1^H‐MRI scans; however, this work used a relatively limited dataset, containing only 45 participants.^17^ Astley et al have demonstrated accurate ^1^H‐MRI segmentation on a large dataset, containing multi‐resolution scans of patients with various pulmonary pathologies.^18^ A 3D UNet was employed and achieved a mean DSC of 0.96 for whole‐lung segmentation across all resolutions.^18^ All these approaches to generate whole‐lung segmentations from ^1^H‐MRI have used single‐channel, mono‐modal CNN‐based methods, where a single image or 3D scan is used as an input to the CNN.^16^, ^17^, ^18^ Although these methods have shown promising results, they cannot account for the aforementioned spatial misalignments between structural and functional modalities. Multichannel approaches using multimodal images have shown promise in DL image analysis applications, where there are important features across multiple imaging modalities.^19^, ^20^ For example, DL has been employed for lesion segmentation using multimodal CT and positron emission tomography (PET) images that are acquired simultaneously.^21^ A similar problem is encountered in this work, thus motivating the investigation of dual‐channel, dual‐modal approaches.

We hypothesized that a dual‐channel approach that leverages both ^1^H‐MRI and Xenon‐129‐MRI (^129^Xe‐MRI) can generate accurate LCEs across a wide range of lung pathologies. We aimed to compare this approach with single‐channel CNN‐based methods, which do not integrate functional and structural imaging as inputs to a CNN. In addition, we aimed to combine the dual‐channel approach with a previously developed DL method for hyperpolarized gas MRI ventilated lung segmentation to generate clinical biomarkers, such as the VDP, without manual intervention.

## Materials and Methods

All prospective studies received ethical approval by the national research ethics committee with participants (or their guardians) providing informed written consent. Appropriate consent and permissions have been granted by the Sponsors to utilize this data for retrospective purposes.

### Patient Data

The dataset included in this study contained 480 corresponding ^1^H‐MRI and ^129^Xe‐MRI scans from 26 healthy participants (median age [range]: 11 [8, 71]; 50% males, 50% females) and 289 patients with various pulmonary pathologies (median age [range]: 47 [6, 83]; 49% males, 51% females). An overview of all participants, stratified by pathology, is displayed in Table 1. The data used in this study were pooled retrospectively from a range of prospective clinical imaging studies.

**TABLE 1:** Summary of Participant Data

Disease	Number of Participants	Number of Scans	Age^a^	Sex^a^	VDP^a^
			Median (range)	Frequency (%)	Median (range)
Asthma	92	154	50 (13, 74)	36 M (40%), 55 F (60%)	2.5 (0.07, 30.9)
Asthma + COPD	25	27	59 (33, 71)	15 M (60%), 10 F (40%)	10.4 (1.3, 29.3)
COPD	20	22	66 (48, 80)	8 M (40%), 12 F (60%)	18.8 (1.9, 64.8)
Cystic fibrosis	55	109	18 (6, 62)	27 M (51%), 26 F (49%)	6.1 (0.38, 62.0)
Healthy	26	27	11 (8, 71)	13 M (50%), 13 F (50%)	0.17 (0.01, 1.6)
ILD^b^	40	71	67 (39, 83)	21 M (58%), 15 F (42%)	7.9 (1.5, 30.1)
Investigation for possible airways disease	15	27	49 (11, 69)	2 M (13%), 13 F (87%)	6.6 (0.65, 35.0)
Preterm birth	42	43	12 (9, 14)	14 M (34%), 27 F (66%)	0.48 (0.01, 5.2)
Total	315	480	44 (6, 83)	138 M (45%), 169 F (55%)	3.6 (0.01, 62.0)

^a^ Demographic information unavailable for eight patients. Age and VDP given at baseline.

^b^ Contains connective tissue disease‐associated interstitial lung disease (CTD‐ILD), hypersensitivity pneumonitis (HP), idiopathic pulmonary fibrosis (IPF) and drug‐induced ILD (DI‐ILD).

COPD = chronic obstructive pulmonary disease; ILD = interstitial lung disease; VDP = ventilated defect percentage; M = male; F = female.

### Image Acquisition

All participants underwent 3D volumetric ^129^Xe‐MRI and ^1^H‐MRI in the coronal plane at approximately functional residual capacity (FRC) + bag (for any given participant, the bag volume was titrated based on standing height and ranges from 400 ml to 1 L) or total lung capacity (TLC) with full lung coverage at 1.5 T on a HDx scanner (GE Healthcare, Milwaukee, WI, USA). A full breakdown of gas doses, titrated based on participant standing height, is included in Supplementary Table S1.

#### ^129^XE‐MRI ACQUISITION

The ^129^Xe was polarized on site to approximately 25% by using an in‐house developed rubidium spin‐exchange polarizer.^22^ Flexible quadrature radiofrequency coils were employed for transmission and reception of MR signals at the Larmor frequency of ^129^Xe‐MRI (Clinical MR Solutions, Brookfield, WI, USA). A 3D balanced steady‐state free precession sequence was used.^23^ The protocol used the following settings: repetition time/echo time of 6.7/2.2 msec, in‐plane resolution of ~4 × 4 m^2^ with a slice thickness of 10 mm. A ~40 cm field of view with a flip angle of 9° or 10° at a bandwidth of ±8 kHz was used.

#### ^1^H‐MRI ACQUISITION

The ^1^H‐MRI scans were acquired with a quadrature transmit–receive body coil in the coronal plane.^23^ A 3D spoiled gradient‐recalled sequence was used with the following settings: repetition time/echo time of 1.9/0.6 msec, in‐plane resolution ~4 × 4 mm^2^ with a slice thickness of 5 mm. A ~40 cm field of view with a flip angle of 5° at a bandwidth of ±83.3 kHz was used. ^1^H‐MRI scans were acquired before and after ^129^Xe‐MRI scans at a similar lung inflation level (i.e. FRC + bag or TLC) and subsequently rigidly registered and resampled to the resolution of ^129^Xe‐MRI, using the ANTs framework implemented in an in‐house MATLAB (Mathworks, Nantucket, MA, USA) software.^24^

### Image Quality Assessment

We determined the prevalence of image artifacts and quantified noise in the testing set to assess their impact on DL performance. Images were classified as either containing or not containing, an artifact for both the ^1^H‐MRI and ^129^Xe‐MRI scans by three blinded expert observers: B.A.T and G.J.C have 10 years and J.R.A has 2 years of experience. Scans would be classified as containing an artifact if the majority of readers scored the scan as containing an artifact. The presence of noise in scans was assessed using the signal‐to‐noise ratio (SNR). Specifically, the SNR was calculated by assessing signal at the trachea and shoulder muscle for ^129^Xe‐MRI and ^1^H‐MRI, respectively. Noise was taken from a random section of the background in each image that did not contain an artifact. This was done to avoid conflating noise with artifacts in the analysis of its impact on DL segmentation performance. Signal and noise were delineated across three consecutive slices for each participant in the testing set. Further details on artifact identification and SNR calculation are provided in Supplementary Figures S1 and S2.

### Lung Cavity Estimation Segmentations

**FIGURE 1:**
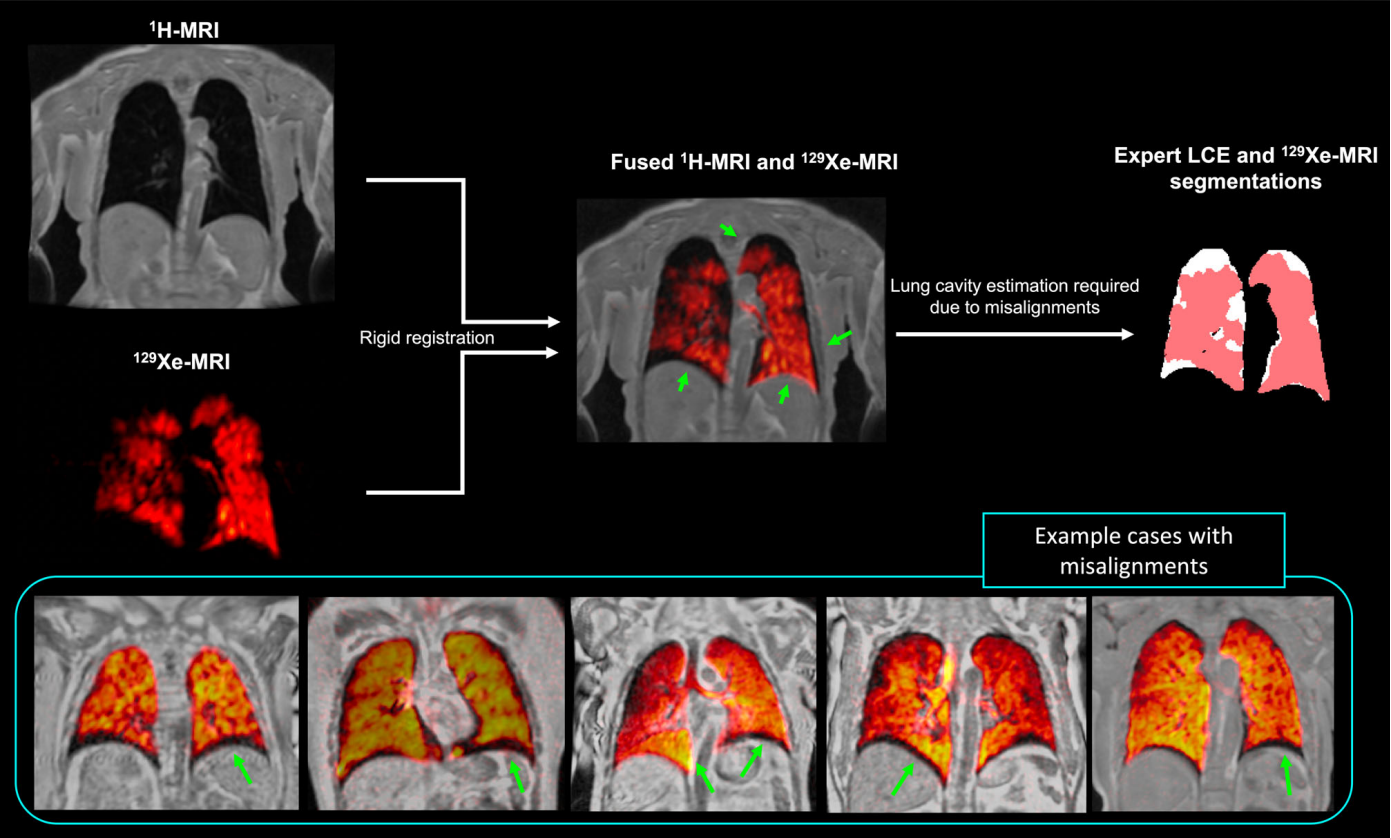
Illustration showing the motivation to generate lung cavity estimations in the spatial domain of ^129^Xe‐MRI due to misalignments in image acquisitions between modalities. Example cases demonstrating misalignments between ^129^Xe‐ and ^1^H‐MRI. Misalignments are indicated by green arrows.

Figure 1 displays fused ^129^Xe‐MRI and ^1^H‐MRI scans after rigid registration, demonstrating the continued misalignment between ventilation and structural scans and thus highlighting the requirement for an LCE. Segmentation of LCEs from ventilation and structural MR image pairs was conducted semi‐automatically using paired spatial fuzzy c‐means clustering (SFCM).^25^ Images are initially bilaterally filtered to remove noise and maintain edges.^26^ The standard FCM algorithm assigns *N* pixels to *C* clusters via fuzzy memberships with the assumption that pixels in close proximity are highly correlated and hence have similarly high membership to the same cluster.^27^ This spatial information will modify the membership value only if, for example, the pixel is noisy and would have been incorrectly classified.

The SFCM method makes use of nearby pixels during the iteration process by considering the membership of voxels within a predefined window and will weigh the central pixel depending on the provided weighting variables.^28^ Heuristic values for the number of clusters and cluster selection threshold for inclusion in the ventilation or structural masks were identified, resulting in the selection of 18 clusters for both masks by A.M.B who had 3.5 years of experience. For the manual segmentations used in this work, the SFCM clustering was applied to both ^129^Xe‐MRI and ^1^H‐MRI scans in a pair‐wise fashion to take advantage of the combined information arising from the co‐location of the image pair.^25^

LCEs were pooled retrospectively from several studies and, consequently, were subsequently manually reviewed and edited by several experienced observers, where each scan was segmented by a single observer, but the dataset as a whole contained LCEs manually edited by observers with a range of expertise: H.M had 7 years, G.J.C had 6 years, P.J.C.H had 5 years, A.M.B had 5 years, L.J.S had 3.5 years, J.A.E had 3 years, and J.R.A had 2 years, of experience in editing LCEs.

### Deep Learning Frameworks

**FIGURE 2:**
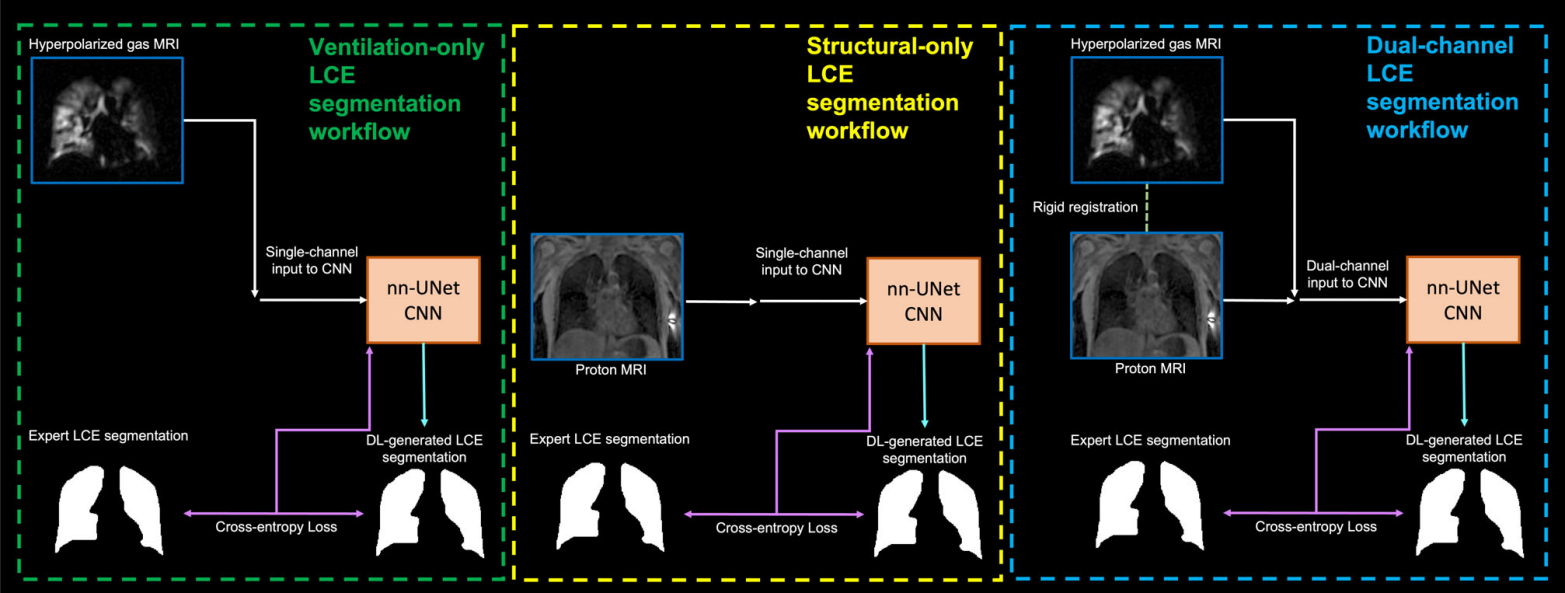
From left to right: ventilation‐only, structural‐only, and dual‐channel deep learning workflows.

We assessed three DL methods to generate LCEs by varying the input channels provided to each network. These consisted of single‐channel and dual‐channel CNN approaches (Fig. 2) as follows:

1. Ventilation‐only (^129^Xe‐MRI)
2. Structural‐only (^1^H‐MRI)
3. Dual‐input (^129^Xe‐MRI + ^1^H‐MRI)

All methods used a variation of the common 2D UNet encoder–decoder network architecture; here, we used a 3D implementation of the UNet, referred to as the nn‐UNet, which has been modified to reduce memory constraints, allowing 30 feature channels.^29^ Convolution operations varied in kernel size from 3 × 3 × 3 to 1 × 1 × 1 depending on the layer of the network. The network also made use of instance normalization. An isotropic spatial window size of 96 × 96 × 96 was used. Each network was trained with a parametric rectified linear unit (PReLU) activation function, Adam optimization, and cross‐entropy loss function. A learning rate of 1 × 10^−5^ and batch size of 2 were used. A decay of 1 × 10^−6^ and L2 regularization were selected to minimize overfitting. Each method was trained for 300 epochs resulting in a model training time of approximately 8 days. All networks were trained using the medical imaging DL framework NiftyNet 0.6.0 (https://github.com/NifTK/NiftyNet) built on top of TensorFlow 1.14.^30^ Training and inference were performed on an NVIDIA Tesla V100 graphical processing unit (GPU) (Nvidia Corporation, Santa Clara, CA, USA) with 16 GB of RAM.

#### DATA AUGMENTATION

Constrained random rotation and scaling were used for data augmentation before ^129^Xe‐MRI and ^1^H‐MRI scans were fed into the network. The augmentation method used does not increase the total size of the dataset but instead utilizes random rotation and scaling factors to modify scans before entering the network. Each time a scan is fed into the network, random rotation and scaling factors with limits −10° to 10° and −10% to 10%, respectively, where different factors at an interval within these limits, were applied.

#### TRAINING AND TESTING SETS

The dataset was divided into training and testing sets; the data split was conducted at the level of scans whereby 15% of the scans were randomly selected as the testing set. If a participant had multiple repeat or longitudinal scans, one scan out of these was randomly selected and the other scans discarded from the analysis; these removed scans do not appear in the dataset. This was done to ensure that no participant was present in both the training and testing sets and that the testing set contained only one scan from each participant, thereby reducing potential biases in favor of specific participants. Therefore, the training set contained 422 corresponding ^129^Xe‐MRI and ^1^H‐MRI scans from a total of 257 participants and the testing set contained 58 scans from 58 participants, representing 81.6% and 18.4% of the total number of participants, respectively. Even though the testing set allocation was randomly determined, at least one scan from each disease or healthy cohort (described in Table 1) was present in the testing set. The training set had the following demographic distributions: median age (range) of 41 (8.9, 83); median VDP (range) 3.23% (0.01, 64.8); sex 44% male, 56% female. The testing set had the following demographic distributions: median age (range) of 53 (6.4, 76); median VDP (range) 5.19% (0.05, 62.0); sex 49% male, 51% female.

### Quantitative Evaluation

#### DICE SIMILARITY COEFFICIENT

The DL‐generated LCEs were evaluated using the overlap‐based DSC metric that assesses the overlap between ground truth (*GT*) and predicted (*PR*) segmentations, defined as:

(1) $$\mathrm{DSC}=2\frac{\left|\mathrm{PR}\cap \mathrm{GT}\right|}{\left|\mathrm{PR}\right|+\left|\mathrm{GT}\right|}$$

#### AVERAGE BOUNDARY HAUSDORFF DISTANCE

Average boundary Hausdorff distance (average HD) in mm is a common distance‐based metric,^31^ which assesses the conformity of boundaries between GTs and PRs and was defined as follows:

(2) $$\mathrm{HD}\left(\mathrm{PR},\mathrm{GT}\right)=\max\left(h\left(\mathrm{PR},\mathrm{GT}\right),h\left(\mathrm{GT},\mathrm{PR}\right)\right)$$

where $h\left(\mathrm{PR},\mathrm{GT}\right)$ represents the directed HD between the sets of PR and GT voxels at the boundary, *pr* represents an individual voxel in the set PR, and *gt* represents an individual boundary voxel in GT. Further, $h\left(\mathrm{PR},\mathrm{GT}\right)$ was defined as:

(3) $$h\left(\mathrm{PR},\mathrm{GT}\right)=\max_{\mathrm{pr}\in \mathrm{PR}}\min_{\mathrm{gt}\in \mathrm{GT}}\left\|\mathrm{PR}-\mathrm{GT}\right\|$$

$\text{where}\left\|\mathrm{PR}-\mathrm{GT}\right\|$ is the Euclidean distance between PR and GT.

#### RELATIVE ERROR METRIC

A relative error metric (XOR) was used to evaluate segmentation errors as follows:

(4) $$\mathrm{XOR}=\frac{\left|\mathrm{PR}\cap {\mathrm{GT}}^{\prime }\right|+\left|{\mathrm{PR}}^{\prime }\cap \mathrm{GT}\right|}{\left|\mathrm{GT}\right|}$$

where PR′ and GT′ are the complements of PR and GT, respectively. The metric was used because it is expected to correlate with the manual editing time required to correct the segmentation outcome.^32^

### Clinical Evaluation

#### LUNG CAVITY ESTIMATION VOLUME

In addition to quantitative evaluation metrics, clinical evaluation metrics were used to assess the lung parenchymal volume defined by the LCE. DL‐generated LCE volumes were compared to ground truth LCE volumes to assess LCE accuracy.

#### VENTILATION DEFECT PERCENTAGE

The VDP has been used as a robust measure of lung function.^7^ VDP was calculated from structural and functional volumes aligned via rigid registration as follows:

(5) $$\text{Ventilation defect percentage}\;\left(\%\right)=\left(1-\frac{\text{ventilated lung volume}}{\mathrm{LCE}\;\text{volume}}\;\right)\times 100\;$$

We assessed the performance of the DL‐generated LCEs by computing VDP values for each scan in the testing set. As shown in Eq 5, in addition to LCE volumes, ventilated lung volumes are required. Thus, we employed a previously trained nn‐UNet fully CNN, developed for automatic hyperpolarized gas MRI ventilated lung segmentation in a large diverse dataset,^33^ which was done to generate accurate DL‐based ^129^Xe‐MRI ventilated lung segmentations for the current testing set. The fully automatic DL‐derived VDPs were compared to VDPs derived from manually edited ventilated and LCE segmentations. Ventilated volumes were initially generated using a binning method.^34^ ^129^Xe‐MRI scans were normalized by the average value of the ^129^Xe signal in the lung cavity and ventilation defects were defined as any value below 33% of the mean signal intensity. Thus, the ventilated volume was defined as the complement of the ventilation defect.^35^

### Statistical Analysis

All statistical analyses were conducted using GraphPad Prism (version 9.2.0; GraphPad Software, San Diego, CA, USA). Data were tested for normality using Shapiro–Wilk tests. When normality was not satisfied, non‐parametric tests were conducted. One‐way repeated measures analysis of variance (ANOVA) or Friedman tests were conducted as appropriate with Bonferroni correction for post hoc multiple comparisons to assess statistical significance of differences between DL ventilation‐only, structural‐only, and dual‐input methods. Pearson or Spearman correlation and Bland–Altman analyses were conducted to compare the volumes of the dual‐input DL method and manual LCEs. In addition, paired t‐tests and Bland–Altman analyses were used to compare manual and DL‐generated VDP values. Independent *t*‐tests with Welch's correction or Mann–Whitney U tests were used as appropriate to assess differences in VDPs between scans containing or not containing artifacts. Relationships between differences in manual and DL‐generated VDPs and SNRs were assessed using Pearson or Spearman correlation. A *P* value < 0.05 was considered statistically significant.

## Results

### Quantitative Evaluation

**FIGURE 3:**
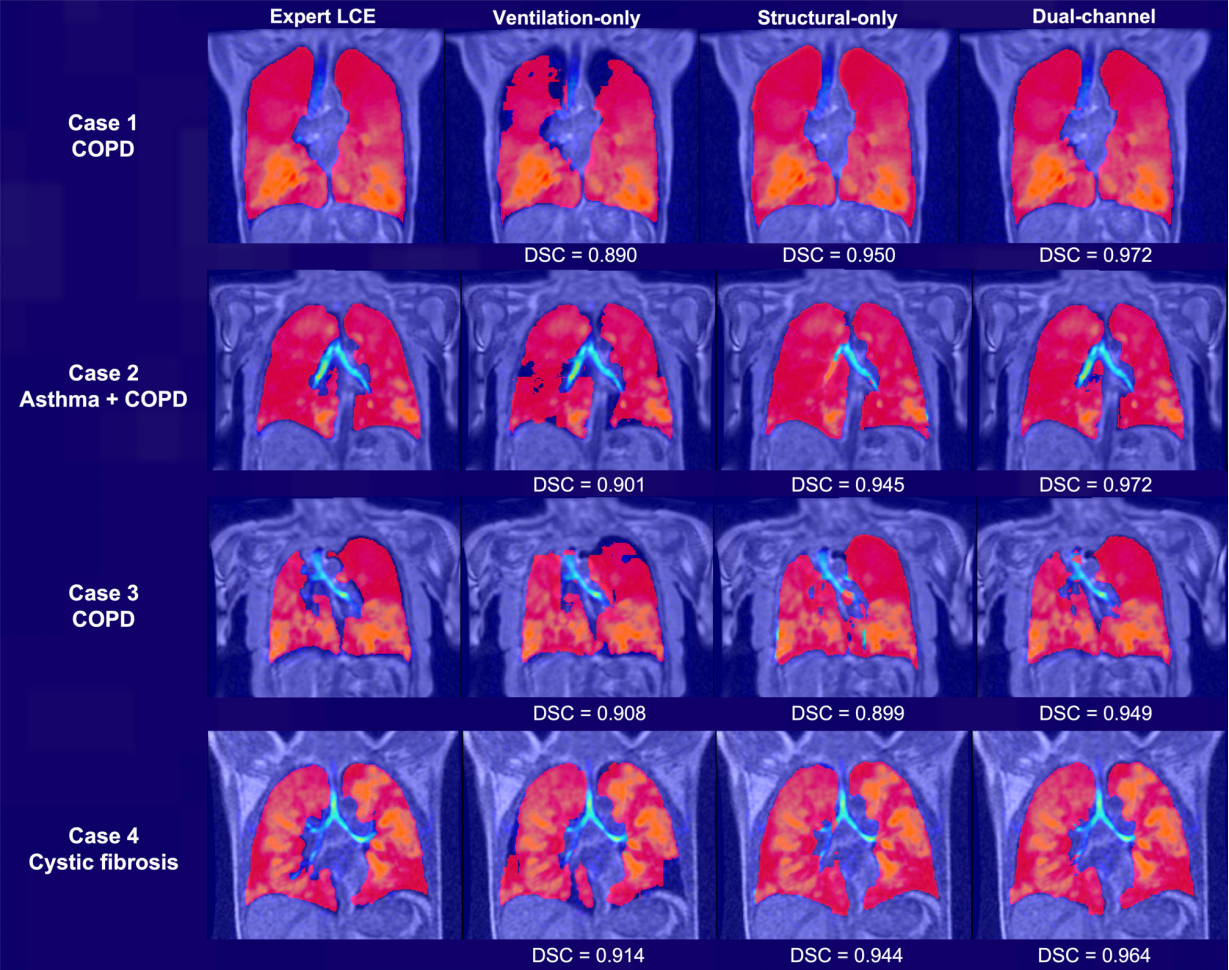
Example coronal slices showing the ^1^H‐MRI fused with the corresponding similar‐breath hyperpolarized gas MRI overlaid with the manual LCE and the LCE generated from the three DL methods for four cases within the testing set. DSC values are provided for each case.

Figure 3 demonstrates the qualitative and quantitative performance of each DL method comparing the DL‐generated LCEs to the manual LCEs for four cases. For all cases, the dual‐input method generated realistic LCEs that might accurately mimic manual LCEs.

**FIGURE 4:**
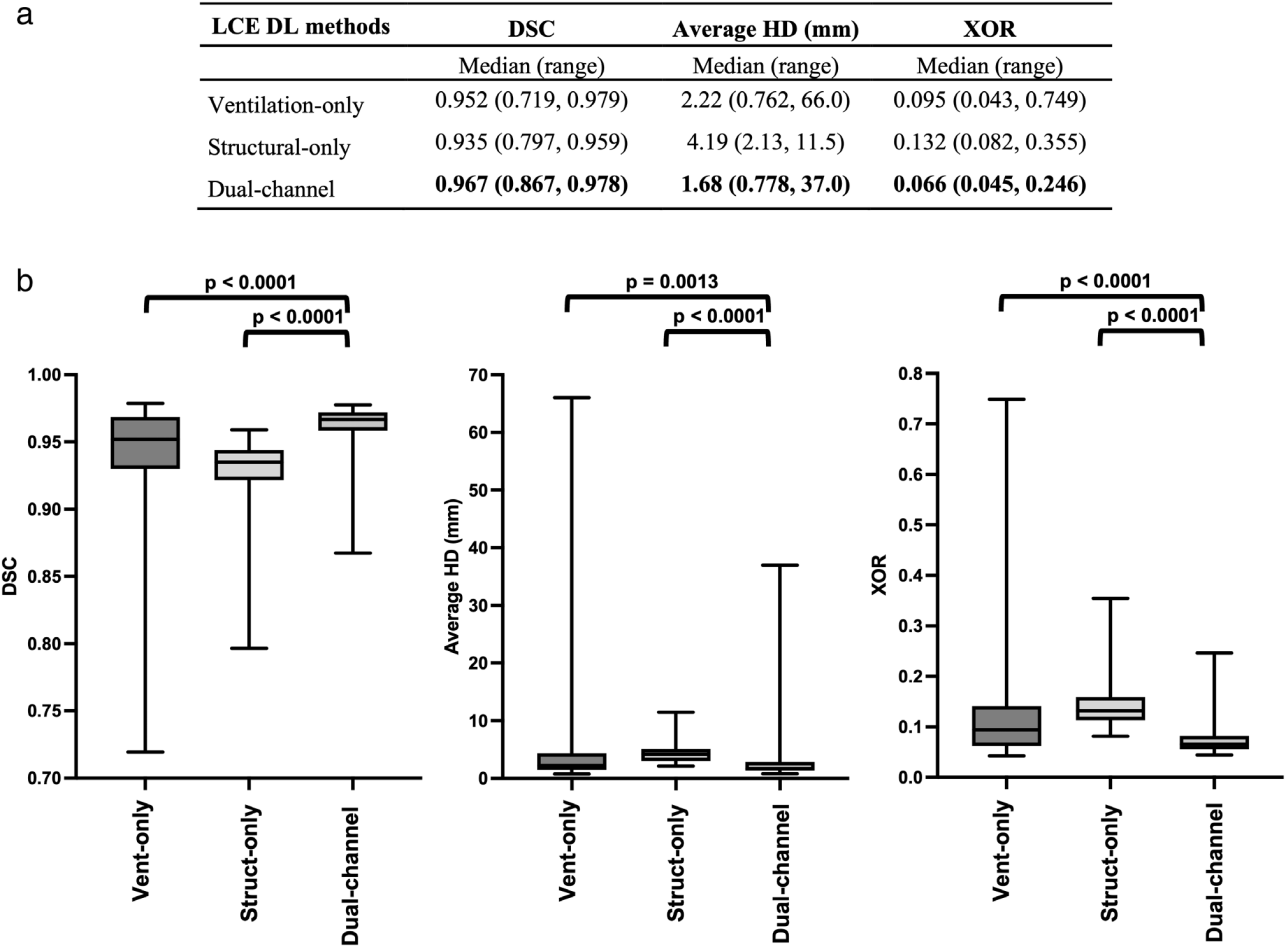
(a) Quantitative results for the testing set (*n* = 58) using the DSC, average HD (mm) and XOR metrics for the ventilation‐only, structural‐only, and dual‐channel DL methods. Median (range) values are given with the best values shown in bold. (b) Comparison of LCE performance for each of the three DL methods using the DSC (left), average HD (center) and XOR (right) metrics. Significance of differences between DL methods as assessed by Friedman tests with Bonferroni correction for multiple comparisons are displayed for each metric.

Quantitative results for each DL method are provided in Fig. 4a. The results demonstrate that the dual‐input method generated the most accurate segmentations across all metrics used. The dual‐input method achieved a median (range) DSC, average HD, and XOR of 0.967 (0.867, 0.978), 1.68 mm (37.0, 0.778 mm), and 0.066 (0.246, 0.045), respectively. The dual‐input method significantly outperformed the single‐channel methods. The results for all metrics are displayed graphically in Fig. 4b. Anterior to posterior segmentation performance is detailed in Supplementary Figure S3. Due to the significant improvements demonstrated by the dual‐input DL method across all segmentation metrics, we selected this method for assessment using clinical evaluation metrics.

### Clinical Evaluation

**FIGURE 5:**
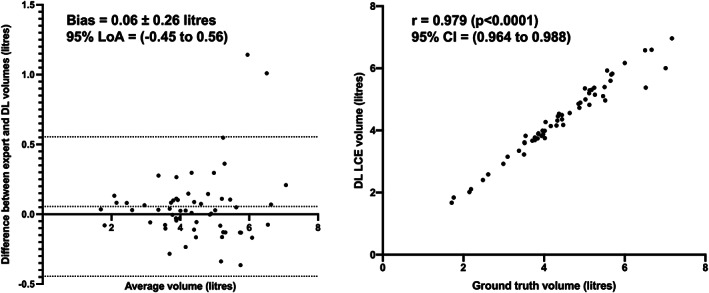
Bland–Altman analysis (left) and Pearson correlation (right) of lung volumes for 58 testing set cases comparing the manual LCEs to the dual‐channel DL‐generated LCEs.

**FIGURE 6:**
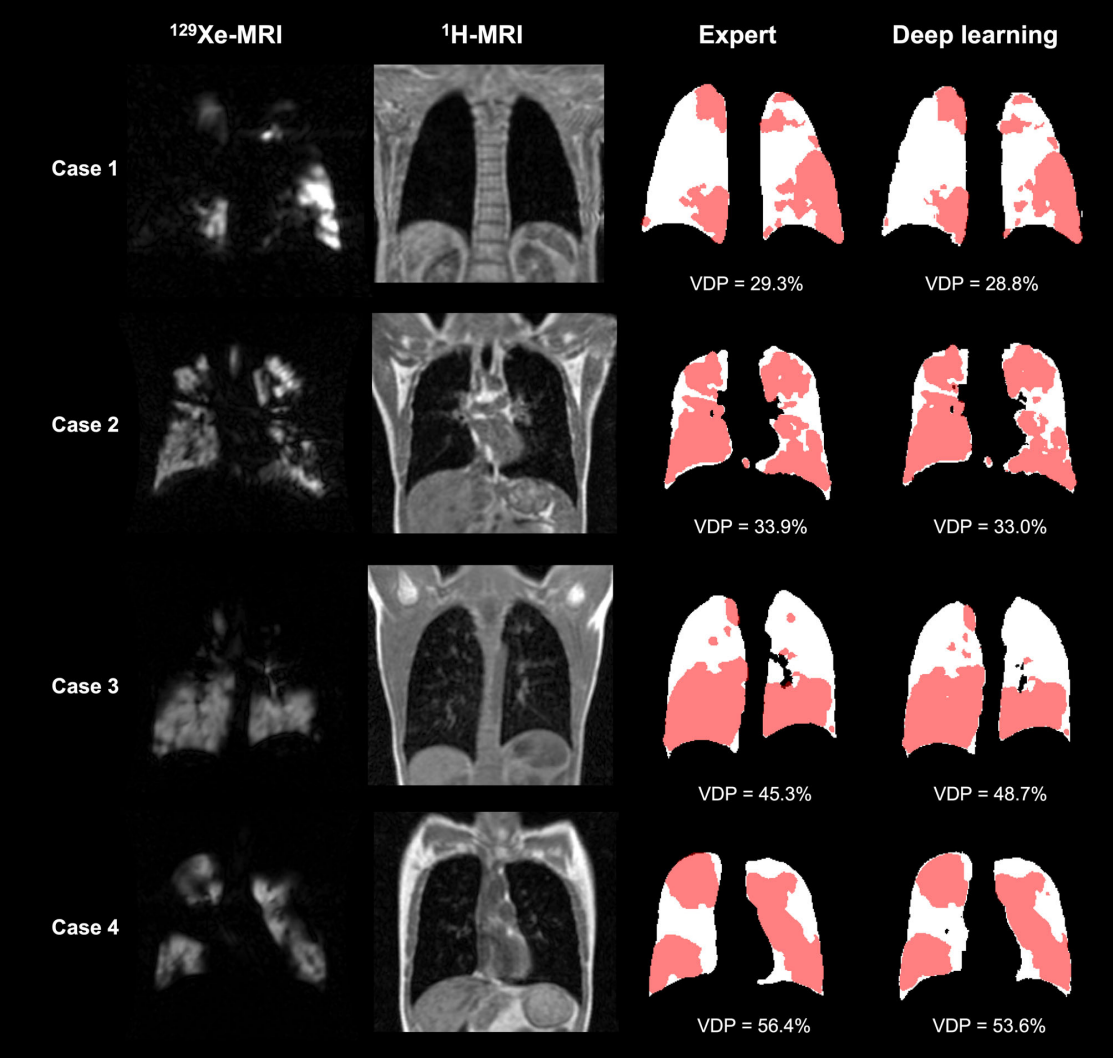
Example coronal slices of four cases with ventilation defects showing fused manual LCEs (white) and hyperpolarized gas MRI ventilated lung segmentations (pink) compared to those generated using the dual‐channel DL method and previously described hyperpolarized gas MRI ventilated lung segmentation method. Manual and DL‐generated VDPs are given for each case.

**FIGURE 7:**
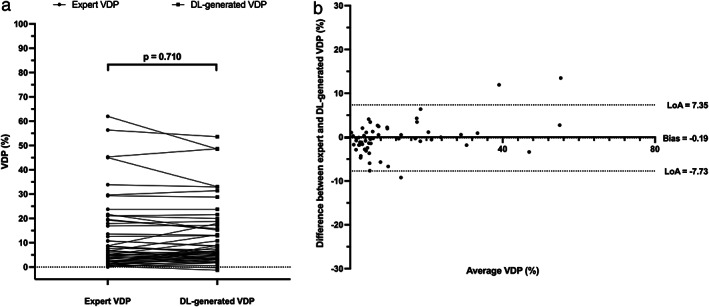
(a) Estimation plot of manual‐ and DL‐generated VDPs (left) with significance of differences; (b) Bland–Altman analysis (right) of VDPs for 58 testing set cases comparing the manual LCE to the dual‐channel DL‐method.

Figure 5 shows Pearson correlation and Bland–Altman analyses of lung volumes for the dual‐input, DL‐generated LCEs compared to manual LCEs. The dual‐input method exhibited a statistically significant, strong Pearson's correlation of 0.98 and minimal bias of 0.06 ± 0.26 liters with limits of agreement (LoA) of −0.45 to 0.56 liters. Figure 6 shows example coronal slices of the manual LCEs and ventilated lung volumes compared with those generated by the DL methods. Figure 7a contains an estimation plot indicating that there is no significant difference between DL‐generated VDPs and manual VDPs (*P* = 0.71). In addition, Bland–Altman analysis of bias using the VDP values resulted in a bias of −0.19% and LoA of −7.73% to 7.35%. A Bland–Altman plot is shown in Fig. 7b for the VDP generated using the proposed DL workflow compared to VDP values from manual assessment.

### Image Quality Assessment

**FIGURE 8:**
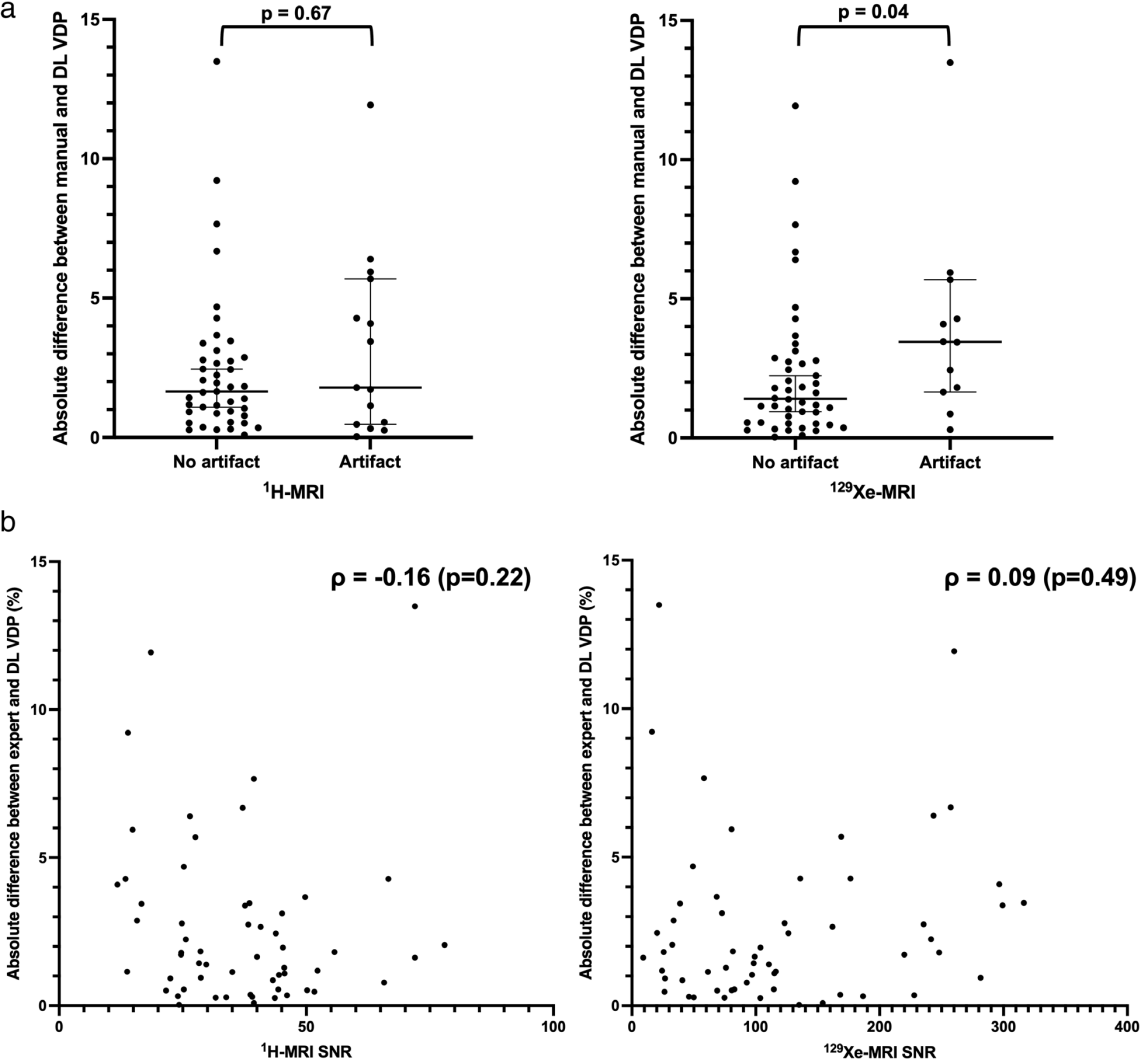
(a) Absolute differences between manual and DL VDPs stratified by presence (or absence) of image artifacts in the ^1^H‐MRI (left) and ^129^Xe‐MRI (right) scans. Mann–Whitney U tests were conducted, and *P* values indicated. (b) Scatterplot of absolute differences between manual and DL VDPs and SNRs for the ^1^H‐MRI (left) and ^129^Xe‐MRI (right) scans. Spearman's *ρ* values are provided.

**FIGURE 9:**
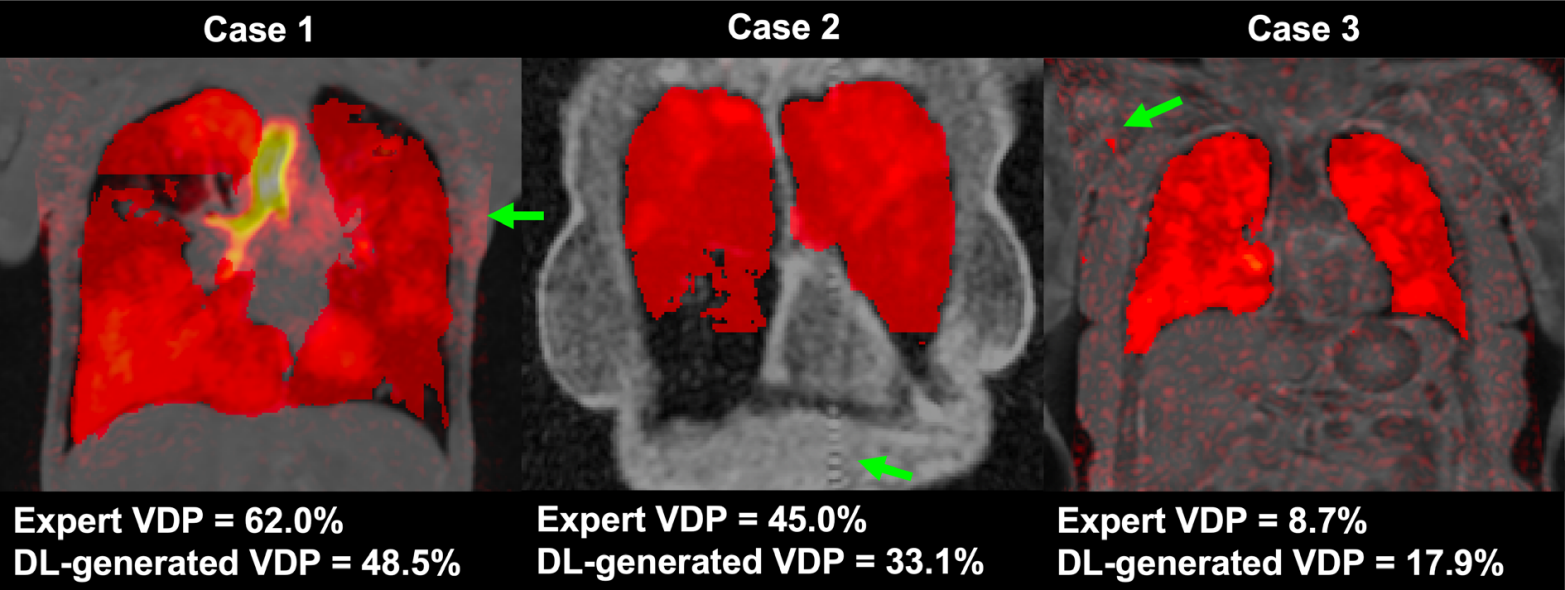
Example coronal slices of fused ^1^H‐MRI, ^129^Xe‐MRI and LCEs from three cases in the testing set which, through Bland–Altman analysis, fall outside the limits of agreement. Manual and DL‐generated VDPs are given. Case 1 contains a motion artifact on the ^129^Xe‐MRI. Case 2 contains a zipper artifact on the ^1^H‐MRI. Case 3 exhibits a large degree of noise throughout the ^129^Xe‐MRI. Artifacts are indicated with green arrows.

In terms of assessing the impact of artifacts in ^1^H‐MRI scans, all three readers agreed on 12 cases and the majority opinion of two readers was used for three cases, resulting in 15 testing set ^1^H‐MRI scans containing an artifact. Nine scans were not included as only one reader identified them as containing an artifact. For ^129^Xe‐MRI scans, all three readers agreed on two cases and the majority opinion of two readers was used for 10 cases, resulting in 12 testing set ^129^Xe‐MRI scans containing an artifact. Thirteen scans were not included as only one reader identified them as containing an artifact. Five cases within the testing set contained artifacts in both ^1^H‐MRI and ^129^Xe‐MRI scans. Artifacts included zipper, aliasing, signal dropout, motion, wrap‐around and image warping. Figure 8a concerns the presence of image artifacts identified by the three independent readers in either the ^1^H‐MRI or ^129^Xe‐MRI scans. The differences between the manual and DL‐generated VDPs were significantly impacted by the presence of imaging artifacts in ^129^Xe‐MRI scans; similar effects were not exhibited when considering artifacts in ^1^H‐MRI scans (*P* = 0.67). Figure 8b plots the Spearman's correlation between the difference in VDP with SNR and shows that there was no significant correlation between the two variables for both ^1^H‐MRI (*P* = 0.22) or ^129^Xe‐MRI (*P* = 0.49) scans. Figure 9 displays three failure cases where the differences in VDP between manual and DL‐generated VDPs are outside the LoA in the Bland–Altman analysis. Case 1 contained a gas motion artifact on the ^129^Xe‐MRI, leading to an error in the segmentation around this region. Case 2 contained a zipper artifact in the ^1^H‐MRI, which traversed the lung parenchyma, possibly contributing to errors in the DL‐generated LCE. Case 3 showed a large degree of noise in the ^129^Xe‐MRI scan.

## Discussion

In this study, we proposed a dual‐channel CNN for LCE that leveraged ^1^H‐MRI and ^129^Xe‐MRI scans. Our method significantly outperformed single‐channel alternatives that do not integrate both functional and structural lung imaging in a range of diseases for adult and pediatric participants. Furthermore, we combined this dual‐channel LCE approach with a DL‐based method for hyperpolarized gas MRI ventilated lung segmentation to automatically generate a key clinical biomarker of lung function, namely, the VDP, showing strong agreement with manually derived VDPs. The proposed method showed no reduction in performance in scans with a large degree of noise; however, it showed decreased performance when artifacts were present in ^129^Xe‐MRI scans.

Qualitative comparison of the various DL methods demonstrated the differences in LCEs due to varying modalities used in the input channels. For the majority of cases, the ventilation‐only method was unable to generate realistic LCEs due to the lack of structural features provided to the CNN. Conversely, the structural‐only method generated reasonable LCEs; however, in cases where there were misalignments between the ^129^Xe‐MRI and ^1^H‐MRI scans, the structural‐only DL method could not account for the inherent registration errors. Misalignments were addressed in the dual‐input method using both ventilation and structural features in the input channels, probably providing the network adequate context to accurately generate LCEs that represented structural lung regions in the domain of ^129^Xe‐MRI. This seems to be supported by the quantitative results adjusted for multiple comparisons, indicating that the dual‐channel method significantly outperformed single‐channel methods across all metrics tested.

The nn‐UNet employed is specifically designed to reduce memory constraints during network training, a requirement that benefits the dual‐channel method, facilitating the use of larger batch and patch sizes.^29^ Previous studies have described DL‐based approaches to segment the lung parenchyma on ^1^H‐MR images; however, these approaches have conducted the segmentation using single‐channel networks.^16^, ^17^, ^18^ The inclusion of functional features present in the hyperpolarized gas MRI scans may provide the network context with which to adapt the structural LCE to account for inherent registration errors between the ^1^H‐MRI and ^129^Xe‐MRI acquisitions. Previous work by Tustison et al utilized separate networks for segmenting ^1^H‐MRI and hyperpolarized gas MRI^16^; however, due to several factors, including inherent registration errors and differences in inflation levels, a network that generates a structural segmentation purely using ^1^H‐MRI seems inadequate.

Although same‐breath acquisition of helium‐3 (^3^He) and ^1^H‐MRI has been leveraged in previous studies,^13^, ^23^, ^36^, ^37^ due to the lower bandwidths and longer repetition times required for ^129^Xe‐MRI, owing to its lower intrinsic signal intensity compared to ^3^He, longer acquisition times and thus longer breath‐holds are inevitable. These are prohibitively long for many patients who are unable to maintain lengthy breath‐holds, inducing movement, particularly at the diaphragm. In this study, ^129^Xe‐MRI was acquired in approximately 10 seconds; ^129^Xe‐/^1^H‐MRI back‐to‐back acquisition times would be approximately 19 seconds. Our recent work with compressed sensing has enabled us to reduce this time to 15 seconds^38^; however, although the shorter breath‐hold is more feasible for patients, the likelihood of changes in lung posture during back‐to‐back scanning persist. As such, a lung cavity estimation will still be required for many patients.

Tustison et al used a 3D UNet CNN to generate ^1^H‐MRI lung segmentations.^16^ However, the authors noted that this limits the batch size due to computational constraints; the nn‐UNet used here may overcome these challenges.^29^ Additionally, the authors generated ventilated lung segmentations of hyperpolarized gas MRI using a 2D CNN.^16^ Conversely, both the dual‐channel DL approach to LCE generation and the single‐channel DL approach to hyperpolarized gas MRI ventilated lung segmentation used here employed 3D CNNs. These 3D CNNs can process images in a fully volumetric fashion. LCEs represent volumetric lung parenchymal regions that are located across multiple slices in the scan; consequently, the network's ability to process scans in three dimensions potentially enhances the delineation of lung parenchymal volumes compared to 2D alternatives, which do not allow the network to learn interslice features of the scan that occur in a volumetric fashion; this has been demonstrated previously in the segmentation of adipose tissue in cardiac MRI.^39^ We used a large, diverse training set comprising patients with numerous pulmonary pathologies and used a testing set that contains only one scan from each participant. This resulted in a robust dual‐channel CNN, which may be demonstrated by the limited bias in the Bland–Altman analysis that showed that the accuracy of the LCEs does not diminish with changing volumes.

Furthermore, evaluation of VDP may demonstrate the ability of DL to both produce accurate LCEs and ventilated lung segmentations. The VDPs generated using the DL workflow exhibited no statistically significant differences with manual VDPs. In addition, the Bland–Altman analysis of VDP showed a bias of only −0.19%. This may indicate that the DL‐generated workflow can provide statistically indistinguishable VDPs without subsequent editing. Removing the editing step could allow for a more streamlined workflow to generate automatic VDP values. This, in turn, leads to a vast reduction in the time taken to generate VDP values. Previous approaches to edit segmentations generated by semi‐automatic segmentation methods could take ~1.5 hours per scan. The automatic DL‐based approach proposed here may eliminate this editing time or could at least drastically reduce it. In addition, inference using the dual‐input method could yield accurate LCEs in ~30 seconds using a single GPU, further facilitating the computation of rapid and robust VDPs, leading to potentially higher clinical throughput.

For all testing set cases, we assessed the impact of SNR and imaging artifacts on DL‐generated VDPs and observed that our approach is potentially invariant to SNR. No significant impact on VDP accuracy was observed due to the presence of at least one artifact (*n* = 15) on the ^1^H‐MRI scans. In contrast, for ^129^Xe‐MRI, there was significantly reduced VDP accuracy for images containing at least one imaging artifact (*n* = 12). This may indicate that the presence of imaging artifacts in ^129^Xe‐MRI scans has the potential to produce inaccurate DL‐generated VDPs, representing a challenge for this approach. The prevalence of imaging artifacts in the training set was not assessed and therefore it cannot be concluded whether the network was exposed to these features previously. In addition, there was less agreement between readers for ^129^Xe‐MRI artifacts, reducing the generalizability of this evaluation.

### Limitations

The large dataset used for this study contained participants with numerous pulmonary pathologies; however, each scan in the dataset is acquired with the same acquisition protocol. This reduced the generalizability of the model as performance has not been demonstrated on scans acquired at a different center, using a different scanner manufacturer, with different field strengths or MRI sequences. Therefore, the proposed DL model is potentially limited in its application to scans acquired with different acquisition protocols. Future investigations will aim to validate approaches on a wider range of scan acquisitions, facilitating intercenter deployment of the proposed DL approach. Nonetheless, we have made our trained model publicly available, which will enable other centers to tailor the model to their unique datasets via the use of fine‐tuning and transfer learning (https://github.com/POLARIS-Sheffield/LCE-segmentation).

While there are multiple examples of good segmentation performances on ^1^H‐MR images with imaging artifacts, the clinical implications of reduced performance on some of these scans is a limitation of our study. Future investigations could employ multiple strategies to reduce the impact of imaging artifacts on DL performance; this could be done by implementing specialized data augmentation techniques such as increasing the proportion of images containing each specific artifact, boosting their prevalence during network training, or by artificially augmenting scans with plausible, synthetic noise. In addition, it may be feasible to build a secondary network to identify the presence of imaging artifacts, hence triggering a manual review; however, there is unlikely to be a sufficiently large dataset to build an effective model for this purpose.

In future work, it may be possible to generate both ventilated and structural lung segmentations within a single model using a dual‐class segmentation network. This approach would have the inherent benefit of co‐location, thereby potentially further dealing with misalignments between imaging modalities. However, the DL‐generated hyperpolarized gas MRI segmentation method used in this work utilized a dataset comprising 759 scans, significantly larger than the dataset used here for LCE; hence, generating ventilated lung segmentations in a dual‐class model would reduce the size of the training set, and consequently likely reduce segmentation performance.

## Conclusion

We used a dual‐channel 3D CNN approach for LCE and compared it to single‐channel DL methods. We demonstrated that the dual‐channel approach, leveraging both hyperpolarized gas and ^1^H‐MRI as inputs, may yield improved LCEs. In addition, we used this approach in conjunction with a DL‐based hyperpolarized gas MRI segmentation method to automatically generate VDPs, which did not significantly differ from manual VDPs.

## Supporting information


**Figure S1** ^129^Xe‐MRI signal (green) and background noise (red) delineations.
**Figure S2.** ^1^H‐MRI signal (green) and background noise (red) delineations.
**Figure S3.** Distribution of DSC values for all 58 cases in the testing set on a slice‐by‐slice basis.
**Table S1.** Titration of ^129^Xe based on standing height.

## References

[1] Vos T , Abajobir AA , Hassen Abate K , et al. Global, regional, and national incidence, prevalence, and years lived with disability for 328 diseases and injuries for 195 countries, 1990‐2016: A systematic analysis for the global burden of disease study 2016. Lancet 2017;390(10100):1211‐1259. 28919117 10.1016/S0140-6736(17)32154-2 PMC5605509

[2] Hollings N , Shaw P . Diagnostic imaging of lung cancer. Eur Respir J 2002;19(4):722‐742. 11999004 10.1183/09031936.02.00280002

[3] Antony J , Carlson DJ , Keall PJ , Xing L . Clinical impact of 4D‐CT imaging on lung cancer radiotherapy treatment planning and biological response. Int J Radiat Oncol Biol Phys 2007;69(3):S526.

[4] Martini K , Frauenfelder T . Emphysema and lung volume reduction: The role of radiology. J Thorac Dis 2018;10(Suppl 23):S2719‐s2731. 30210824 10.21037/jtd.2018.05.117 PMC6129802

[5] Eichinger M , Heussel C‐P , Kauczor H‐U , Tiddens H , Puderbach M . Computed tomography and magnetic resonance imaging in cystic fibrosis lung disease. J Magn Reson Imaging 2010;32(6):1370‐1378. 21105141 10.1002/jmri.22374

[6] Wild JM , Marshall H , Bock M , et al. MRI of the lung (1/3): Methods. Insights Imaging 2012;3(4):345‐353. 22695952 10.1007/s13244-012-0176-x PMC3481083

[7] Woodhouse N , Wild JM , Paley MN , et al. Combined helium‐3/proton magnetic resonance imaging measurement of ventilated lung volumes in smokers compared to never‐smokers. J Magn Reson Imaging 2005;21(4):365‐369. 15779032 10.1002/jmri.20290

[8] Horn FC , Marshall H , Collier GJ , et al. Regional ventilation changes in the lung: Treatment response mapping by using hyperpolarized gas MR imaging as a quantitative biomarker. Radiology 2017;284(3):854‐861. 28471738 10.1148/radiol.2017160532

[9] Tahir BA , Bragg CM , Wild JM , et al. Impact of field number and beam angle on functional image‐guided lung cancer radiotherapy planning. Phys Med Biol 2017;62(17):7114‐7130. 28800298 10.1088/1361-6560/aa8074

[10] Fain SB , Korosec FR , Holmes JH , O'Halloran R , Sorkness RL , Grist TM . Functional lung imaging using hyperpolarized gas MRI. J Magn Reson Imaging 2007;25(5):910‐923. 17410561 10.1002/jmri.20876

[11] Hughes PJC , Smith L , Chan H‐F , et al. Assessment of the influence of lung inflation state on the quantitative parameters derived from hyperpolarized gas lung ventilation MRI in healthy volunteers. J Appl Physiol (Bethesda, MD: 1985) 2019;126(1):183‐192. 10.1152/japplphysiol.00464.2018 PMC6383640 30412033

[12] Stewart NJ , Smith LJ , Chan HF , et al. Lung MRI with hyperpolarised gases: Current & future clinical perspectives. Br J Radiol 2021;95:20210207. 34106792 10.1259/bjr.20210207 PMC9153706

[13] Tahir BA , Swift AJ , Marshall H , et al. A method for quantitative analysis of regional lung ventilation using deformable image registration of CT and hybrid hyperpolarized gas/1H MRI. Phys Med Biol 2014;59(23):7267‐7277. 25383657 10.1088/0031-9155/59/23/7267

[14] Gerard SE , Herrmann J , Kaczka DW , Musch G , Fernandez‐Bustamante A , Reinhardt JM . Multi‐resolution convolutional neural networks for fully automated segmentation of acutely injured lungs in multiple species. Med Image Anal 2020;60:101592. 31760194 10.1016/j.media.2019.101592 PMC6980773

[15] Astley JR , Wild JM , Tahir BA . Deep learning in structural and functional lung image analysis. Br J Radiol 2020;95:20201107. 10.1259/bjr.20201107 PMC9153705 33877878

[16] Tustison NJ , Avants BB , Lin Z , et al. Convolutional neural networks with template‐based data augmentation for functional lung image quantification. Acad Radiol 2019;26(3):412‐423. 30195415 10.1016/j.acra.2018.08.003 PMC6397788

[17] Zha W , Fain SB , Schiebler ML , Evans MD , Nagle SK , Liu F . Deep convolutional neural networks with multiplane consensus labeling for lung function quantification using UTE proton MRI. J Magn Reson Imaging 2019;50(4):1169‐1181. 30945385 10.1002/jmri.26734 PMC7039686

[18] Astley JR , Biancardi A , Marshall H , et al. Generalizable deep learning for multi‐resolution proton MRI lung segmentation in multiple diseases. Proceedings of the 29th Annual Meeting of ISMRM. Volume (abstract 3224). Online. International Society for Magnetic Resonance in Medicine; 2021.

[19] Xu Y . Deep learning in multimodal medical image analysis. International conference on health information science. Berlin, Heidelberg: Springer; 2019. p 193‐200.

[20] Yang X , Tjio G , Yang F , et al. A multi‐channel deep learning approach for segmentation of the left ventricular endocardium from cardiac images. 2019 41st annual international conference of the IEEE engineering in medicine and biology society (EMBC). Berlin, Germany: IEEE; 2019. p 4016‐4019. 10.1109/EMBC.2019.8856833 31946752

[21] Guo Z , Li X , Huang H , Guo N , Li Q . Deep learning‐based image segmentation on multimodal medical imaging. IEEE Trans Radiat Plasma Med Sci 2019;3(2):162‐169. 34722958 10.1109/trpms.2018.2890359 PMC8553020

[22] Norquay G , Collier GJ , Rao M , Stewart NJ , Wild JM . Xe^129^‐Rb spin‐exchange optical pumping with high photon efficiency. Phys Rev Lett 2018;121(15):153201. 30362785 10.1103/PhysRevLett.121.153201

[23] Stewart NJ , Chan HF , Hughes PJC , et al. Comparison of (3) He and (129) Xe MRI for evaluation of lung microstructure and ventilation at 1.5T. J Magn Reson Imaging 2018;48(3):632‐642. 29504181 10.1002/jmri.25992 PMC6175321

[24] Avants BB , Tustison NJ , Stauffer M , Song G , Wu B , Gee JC . The insight ToolKit image registration framework. Front Neuroinform 2014;8:44. 24817849 10.3389/fninf.2014.00044 PMC4009425

[25] Biancardi A , Acunzo L , Marshall H , et al. A paired approach to the segmentation of proton and hyperpolarized gas MR images of the lungs. Proceedings of the 26th Annual Meeting of ISMRM. Volume (abstract 2442). Paris: ISMRM; 2018.

[26] Tomasi C , Manduchi R . Bilateral filtering for gray and color images. Sixth international conference on computer vision (cat No 98 CH36271). Mumbai, India: IEEE; 1998. p 839‐846.

[27] Bezdek JC , Ehrlich R , Full W . FCM: The fuzzy c‐means clustering algorithm. Comput Geosci 1984;10(2):191‐203.

[28] Chuang K‐S , Tzeng H‐L , Chen S , Wu J , Chen T‐J . Fuzzy c‐means clustering with spatial information for image segmentation. Comput Med Imaging Graph 2006;30(1):9‐15. 16361080 10.1016/j.compmedimag.2005.10.001

[29] Isensee F , Petersen J , Klein A , et al. nnu‐net: Self‐adapting framework for u‐net‐based medical image segmentation; 2018. arXiv preprint arXiv:180910486.

[30] Gibson E , Li W , Sudre C , et al. NiftyNet: A deep‐learning platform for medical imaging. Comput Methods Programs Biomed 2018;158:113‐122. 29544777 10.1016/j.cmpb.2018.01.025 PMC5869052

[31] Shapiro MD , Blaschko MB . On Hausdorff distance measures. MA: Computer Vision Laboratory University of Massachusetts Amherst; 2004. p 1003.

[32] Biancardi AM , Wild JM . New disagreement metrics incorporating spatial detail – Applications to lung imaging. In: Valdés Hernández M , González‐Castro V , editors. Medical image understanding and analysis. Cham: Springer International Publishing; 2017. p 804‐814.

[33] Astley JR , Biancardi AM , Hughes PJC , et al. Large‐scale investigation of deep learning approaches for ventilated lung segmentation using multi‐nuclear hyperpolarized gas MRI. Sci Rep 2022;12(1):10566. 35732795 10.1038/s41598-022-14672-2 PMC9217976

[34] He M , Kaushik SS , Robertson SH , et al. Extending semiautomatic ventilation defect analysis for hyperpolarized (129)Xe ventilation MRI. Acad Radiol 2014;21(12):1530‐1541. 25262951 10.1016/j.acra.2014.07.017 PMC4254215

[35] Collier GJ , Acunzo L , Smith LJ , et al. Linear binning maps for image analysis of pulmonary ventilation with hyperpolarized gas MRI: transferability and clinical applications. Proceedings of the 26th Annual Meeting of ISMRM. Volume (abstract 4482). Paris: ISMRM; 2018.

[36] Wild JM , Ajraoui S , Deppe MH , et al. Synchronous acquisition of hyperpolarised 3He and 1H MR images of the lungs ‐ maximising mutual anatomical and functional information. NMR Biomed 2011;24(2):130‐134. 20821726 10.1002/nbm.1565

[37] Tahir BA , Hughes PJC , Robinson SD , et al. Spatial comparison of CT‐based surrogates of lung ventilation with hyperpolarized Helium‐3 and Xenon‐129 gas MRI in patients undergoing radiation therapy. Int J Radiat Oncol Biol Phys 2018;102(4):1276‐1286. 30355463 10.1016/j.ijrobp.2018.04.077

[38] Collier GJ , Hughes PJC , Horn FC , et al. Single breath‐held acquisition of coregistered 3D 129Xe lung ventilation and anatomical proton images of the human lung with compressed sensing. Magn Reson Med 2019;82(1):342‐347. 30821003 10.1002/mrm.27713

[39] Kulasekara M , Dinh VQ , Fernandez‐del‐Valle M , Klingensmith JD . Comparison of two‐dimensional and three‐dimensional U‐net architectures for segmentation of adipose tissue in cardiac magnetic resonance images. Med Biol Eng Comput 2022;60:2291‐2306. 35726000 10.1007/s11517-022-02612-1 PMC11321535

