# Supplementary material for: A Dual‐Channel Deep Learning Approach for Lung Cavity Estimation From Hyperpolarized Gas and Proton MRI
Source: J Magn Reson Imaging. 2022 Nov 14;57(6):1878–90. doi: 10.1002/jmri.28519 (PMC10947587; doi:10.1002/jmri.28519)
Supplement: Supplementary file 1 — Figure S1 129Xe‐MRI signal (green) and background noise (red) delineations. Figure S2. 1H‐MRI signal (green) and background noise (red) delineations. Figure S3. Distribution of DSC values for all 58 cases in the testing set on a slice‐by‐slice basis. Table S1. Titration of 129Xe based on standing height. [file JMRI-57-1878-s001.docx]

**Supplementary material 1**

***Artifact analysis***

Testing set scans were classified as either containing, or not containing, an artifact for both the ^1^H-MRI and ^129^Xe-MRI scans. An image was classified as containing an artifact if and only if the artifact was inside the lung parenchyma or within the region encompassed by the ribs. This was chosen to focus solely on artifacts that were likely to have a significant impact on DL-based LCE performance. Artifacts were determined by three readers; B.A.T and G.J.C have 10 years and J.R.A has 2 years of experience. B.A.T and G.J.C are both imaging scientists with extensive experience in the pulmonary MRI field and J.R.A is currently undertaking a Ph.D. in lung imaging. Each reader was blinded, and ^129^Xe-MRI and ^1^H-MRI scans were assessed over two sessions. ^1^H-MRI scans were assessed for artifacts initially followed by ^129^Xe-MRI scans with a washout period of 24 hours for J.R.A and G.J.C; B.A.T performed the analysis similarly but with a 4-hour washout period between sessions. Scans would be classified as containing an artifact if the majority of readers scored the scan as containing an artifact.

***SNR analysis and calculation***

We determined the SNR for all testing set cases in the ^129^Xe-MRI and ^1^H-MRI scans in order to assess the impact of noise on the performance of DL-based LCEs. SNR was calculated as follows:

$$SNR= \frac{Mean signal itensity}{Standard deviation of noise}$$

*^129^Xe-MRI SNR analysis*

Signal was assessed at a high signal location within the trachea and noise was delineated in two locations for each slice, one under the diaphragm and the other above the apex. It was ensured that both the noise and signal were calculated on regions not containing an artifact as to not conflate the artifact and SNR analyses of DL segmentation performance. Signal and noise were delineated on the central slice of the scan and one slice either side of the central slice, resulting in three consecutive slices being delineated for each participant. Figure S1 shows the central slice signal and noise delineations for nine random cases in the testing set.


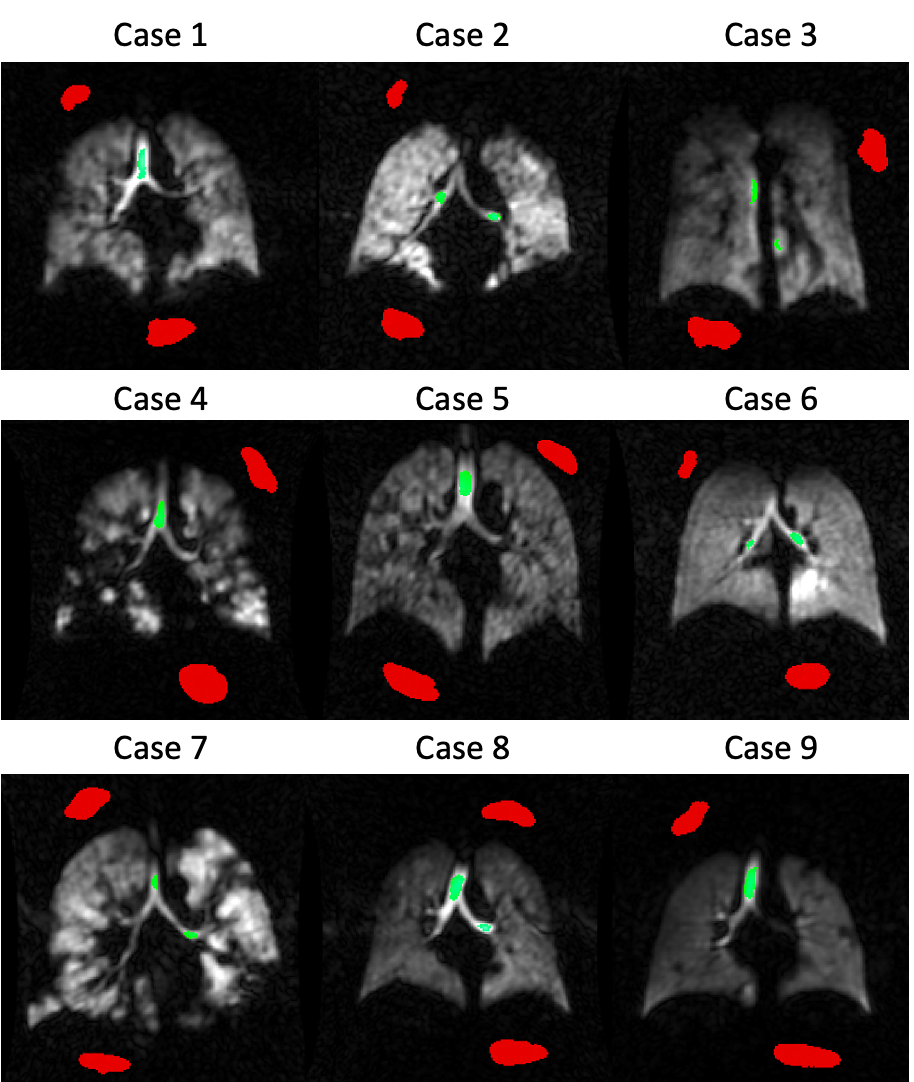


Figure S1. ^129^Xe-MRI signal (green) and background noise (red) delineations.

*^1^H-MRI SNR analysis*

Signal was assessed at a location within the shoulder muscle and noise was delineated outside of the chest cavity. It was ensured that both the noise and signal were calculated on regions not containing an artifact as to not conflate these two analyses of DL segmentation performance. Signal and noise were delineated on the central slice of the scan and one slice either side of the central slice, resulting in three consecutive slices being delineated for each participant. Figure S2 shows the central slice signal and noise delineations for nine random cases in the testing set.


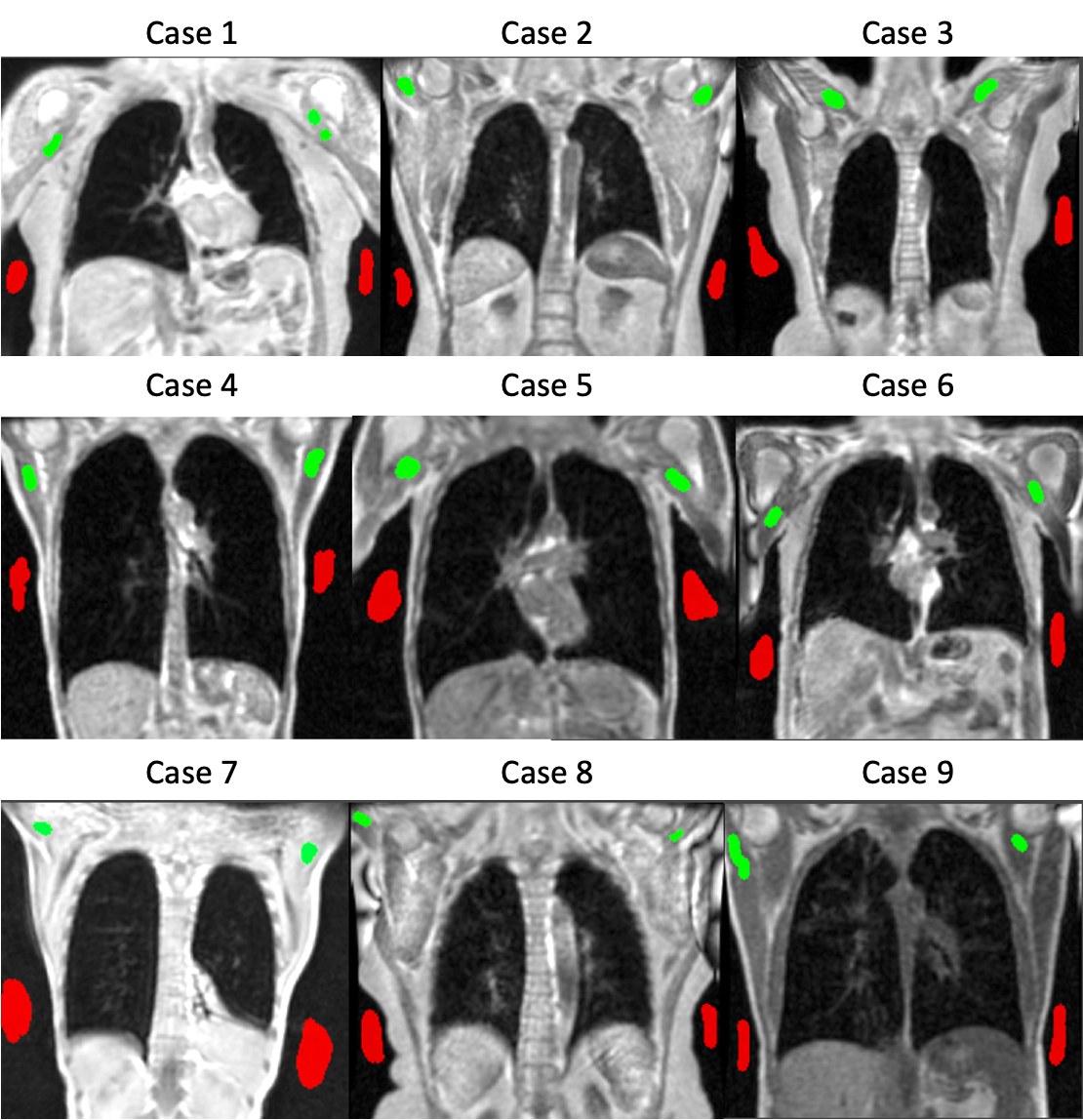


Figure S2. ^1^H-MRI signal (green) and background noise (red) delineations.

**Supplementary material 2**

***Calculation of bag volume titrated based on participant height***

Bag volume (whether the image acquired was FRC+bag or TLC) was always titrated based on standing height, resulting in bag volumes between 400mL and 1L. This bag volume for any given patient was then matched if they performed FRC+bag and TLC manoeuvres at the same session. For the FRC+bag images, the patients held their breath after inhaling the bag. For the TLC images, the patients first inhaled the bag, then immediately carried on inhaling room air until TLC was reached - then held their breath. The sole difference between the FRC and TLC bags was the concentration of the inhaled gases. Table S1 below demonstrates how the bag volume was titrated based on standing height.

Table S1. Titration of ^129^Xe based on standing height.

**Supplementary material 3**

***LCE segmentation performance on anterior to posterior slices***

Figure S3 shows the distribution of DSC values per slice (anterior to posterior) on slices containing the lung parenchyma for all 58 scans in the testing set. LCE accuracy did not reduce substantially in posterior or anterior slices; however, in some cases where there was partial voluming on the final slice, the DL-based LCE frequently segmented this region whereas manual segmentations often excluded it. Voxel-based evaluation metrics are known to increase in sensitivity when the number of voxels is reduced; hence, on the initial and final slices of the lung cavity, where there are only small numbers of voxels in the expert segmentations, the performance is reduced in terms of DSC.

**
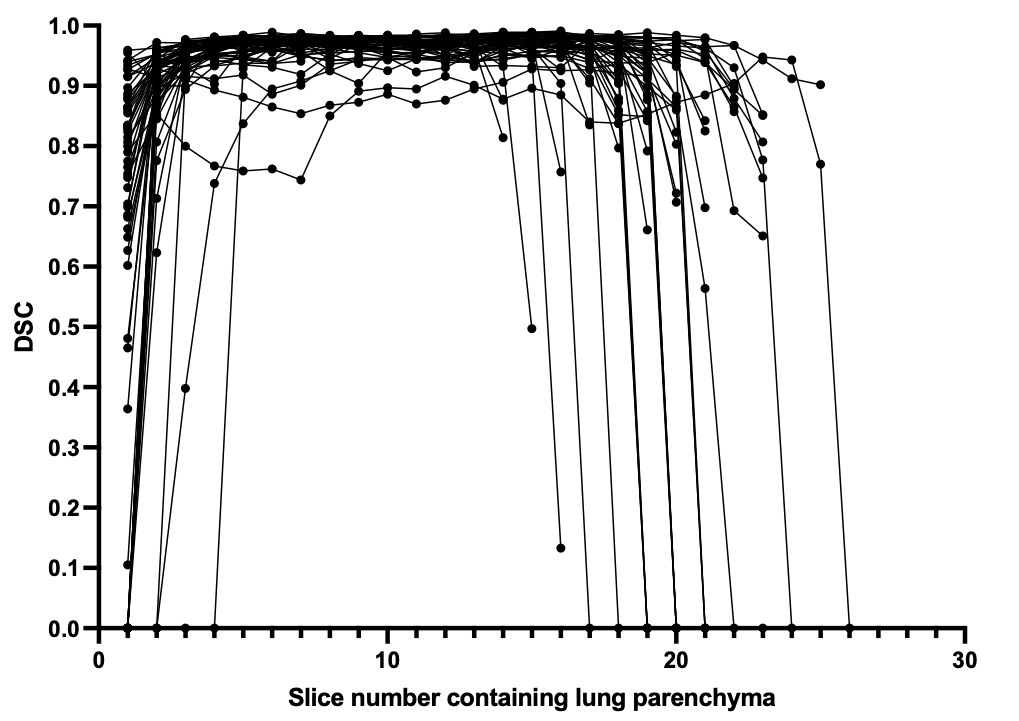
**

Figure S3. Distribution of DSC values for all 58 cases in the testing set on a slice-by-slice basis.
